# Risk Factors for Mortality among 2009 A/H1N1 Influenza Hospitalizations in Maricopa County, Arizona, April 2009 to March 2010

**DOI:** 10.1155/2012/914196

**Published:** 2012-07-15

**Authors:** G. Chowell, A. Ayala, V. Berisha, C. Viboud, M. Schumacher

**Affiliations:** ^1^School of Human Evolution and Social Change, Arizona State University, Tempe, AZ 85287, USA; ^2^Division of Epidemiology and Population Studies, Fogarty International Center, National Institutes of Health, Bethesda, MD 20892, USA; ^3^Office of Epidemiology, Maricopa County Department of Public Health, Phoenix, AZ 85012, USA

## Abstract

We analyzed individual-level data on pandemic influenza A/H1N1pdm hospitalizations from the enhanced surveillance system of the Maricopa County Department of Public Health, AZ, USA from April 1st, 2009 to March 31st, 2010. We also assessed the the risk of death among A/H1N1 hospitalizations using multivariate logistic regression. Hospitalization rates were significantly higher among Native Americans (risk ratio (RR) ** **= ** **6.2; 95% CI: 6.15, 6.21), non-Hispanic Black (RR = 3.84; 95% CI: 3.8, 3.9), and Hispanics (RR = 2.0; 95% CI: 2.0, 2.01) compared to non-Hispanic Whites. Throughout the spring, 59.2% of hospitalized patients received antiviral treatment; the proportion of patients treated increased significantly during the fall to 74.4% (Chi-square test, *P* < 0.0001). In our best-fit logistic model, the adjusted risk of death among A/H1N1 inpatients was significantly higher during the fall wave (August 16, 2009 to March 31, 2010, OR = 3.94; 95% CI: 1.72, 9.03) compared to the spring wave (April 1, 2009 to August 15, 2009). Moreover, chronic lung disease (OR = 3.5; 95% CI: 1.7, 7.4), cancer within the last 12 months (OR = 4.3; 95%CI: 1.3, 14.8), immuno-suppression (OR = 4.0; 95% CI: 1.84, 8.9), and admission delays (OR = 4.6; 95% CI: 2.2, 9.5) were significantly associated with an increased the risk of death among A/H1N1 inpatients.

## 1. Introduction

The first cases of the 2009 A/H1N1 influenza pandemic were confirmed in California on April 21, and in Mexico on April 23, 2009 [1]. In the state of Arizona, the first case of novel A/H1N1 influenza was confirmed on April 29 and the first death associated with the novel A/H1N1 virus was identified on May 14th with a date of illness onset on April 28, 2009. Preliminary estimates of the 2009 A/H1N1 influenza pandemic burden indicate that between 7,500 and 44,100 deaths can be attributed to the novel A/H1N1 influenza virus in the United States (US) from May through December, 2009 [2].

In Maricopa County (MC), AZ the first wave of novel A/H1N1 started in late April 2009, closely following the first detection of the virus in California. A second wave of illness began around August 2009 and peaked in October 2009. At the beginning of the first wave, the Department of Public Health (MCDPH) Office of Epidemiology put in place an enhanced surveillance system to identify inpatients diagnosed with 2009 A/H1N1 influenza across all hospitals in the county. The rapid increase of novel A/H1N1 influenza cases at an unusual time of the year prompted the MCDPH to enhance surveillance activities, increase communication with local healthcare providers, establish collaborations with state and federal public health agencies, and disseminate continuous updates on the pandemic status to the community.

Analyzing the impact of the 2009 A/H1N1 influenza in MC is of particular interest as 39% of the population is composed of Hispanics, non-Hispanic Blacks, Native Americans, and Asians. Assessing differences in hospitalization and death rates according to ethnic/race groups could inform preventive and control efforts by helping identify vulnerable populations at increased risk of severe disease outcomes. Thus, we analyzed individual-level data on hospitalized patients with laboratory-confirmed A/H1N1pdm influenza complied by the enhanced surveillance system put in place by MCDPH from April 1, 2009 to March 31, 2010. This type of study could shed light on the identification of vulnerable subpopulations at increased risk of severe disease outcomes and inform prevention guidelines for epidemic and pandemic influenza.

## 2. Materials and Methods

### 2.1. The Study Location: Maricopa County

Maricopa County is the third most populous local public health jurisdiction in the US, behind New York City and Los Angeles County, with a population of 3.8 million comprising 60% percent of Arizona state's population. 

### 2.2. Epidemiological and Population Data

Detailed data on hospitalized patients with A/H1N1 influenza was available from an enhanced epidemiological surveillance system that was put in place to keep track of the 2009 influenza pandemic by the MCDPH Office of Epidemiology. Enhanced surveillance was conducted at all hospitals in MC to detect patients hospitalized with confirmed 2009 A/H1N1 infection and to detect A/H1N1 deaths. MCDPH requested that all Maricopa County hospitals consider for testing patients presenting with fever (>37.8°C or 100°F) and respiratory symptoms (including cough and sore throat) or sepsis-like syndrome. Medical records and laboratory results were reviewed as cases were reported to the surveillance system. Information collected on a standard form included demographics (age, gender, ethnicity/race), dates of onset of symptoms and hospitalization, underlying risk factor data (asthma, chronic lung disease, cardiac disease, obesity, metabolic disease, diabetes, kidney disease, cancer during the last 12 months, immunosuppression, and neurological disease), hospitalization duration, and whether the patient was treated with neuraminidase inhibitors. Immunosuppressive conditions included patients undergoing chemotherapy, chronic corticosteroid therapy, immunosuppressant therapy, or patients diagnosed with DiGeorge Syndrome, Wiskott-Aldrich Syndrome, HIV/AIDS, hypogammaglobulinemia, and organ transplant recipients. We defined the admission delay as the time elapsed from symptoms onset to hospitalization admission. We stratified admission delay into two groups ≤2 and >2 days based on treatment recommendations to initiate antiviral medication within 48 h of disease onset. Antiviral treatment with neuraminidase inhibitors (Oseltamivir and Zanamivir) was considered for all hospitalized patients with suspected A/H1N1 infection. In addition, MCDPH developed a comprehensive surveillance methodology to identify A/H1N1 deaths. This effort included close collaboration with the MC Office of the Medical Examiner (ME) to investigate all suspect A/H1N1 deaths and with the Office of Vital Statistics by cross-referencing death certificates with cases reported to MCDPH. 

Our analysis is focused on patients diagnosed with 2009 A/H1N1 influenza who were hospitalized in public and private hospitals in MC from April 2009 to March 2010 and for which a standard investigation form was completed. Laboratory diagnosis of patients was performed by RT-PCR or viral culture. Hospitalizations for which a form was not completed were excluded from analysis. Based on the date of notification, the first pandemic wave (spring wave) comprised the period from April 1, 2009 to August 15, 2009 while the fall pandemic wave was from August 16, 2009 to March 31, 2010 and coincided with the return of students from summer vacations. 

We also calculated standardized hospitalization rates based on population estimates according to age and race groups for MC in 2009 [3].

### 2.3. Hospitalized Case Fatality Ratios 

The hospitalized case fatality ratio (hCFR) measures the severity of an infectious disease and is defined as the proportion of deaths among all hospitalized cases. Here, we estimated the case fatality ratio among novel A/H1N1 hospitalizations (HCFR = H1N1 inpatient deaths/H1N1 inpatients). We analyzed the hCFR according to administration of neuraminidase inhibitor, age groups, ethnic/race groups, and pandemic waves.

### 2.4. Modeling Risk of Death among A/H1N1 Influenza Hospitalizations

We used multivariate logistic regression models to assess the risk of A/H1N1 death among hospitalized patients with A/H1N1 influenza after adjustment for age, gender, ethnicity/race, pandemic wave, admission delay, antiviral treatment, and underlying medical conditions. In particular, we sought to investigate the effect of ethnicity/race among Hispanics, non-Hispanic Whites, non-Hispanic Blacks, Asians, and Native Americans on the risk of death among A/H1N1 inpatients. We also quantified the interaction between antiviral treatment and admission delay to account for the fact that patients admitted earlier in their disease course had a higher probability of receiving antiviral treatment. We analyzed fully adjusted and simplified logistic regression models generated via stepwise backward elimination. We measured the strength of model predictors using odds ratios (95% CI) and *P* values. Records with missing data were excluded from the analysis.

This study was granted exempt IRB status from Arizona State University (IRB Protocol number: 1205007823). All investigations were part of public health practice; all individual data were kept confidential, and patients could not be identified. Statistical analyses were performed using SPSS 20.0.

## 3. Results

**Figure 1:**
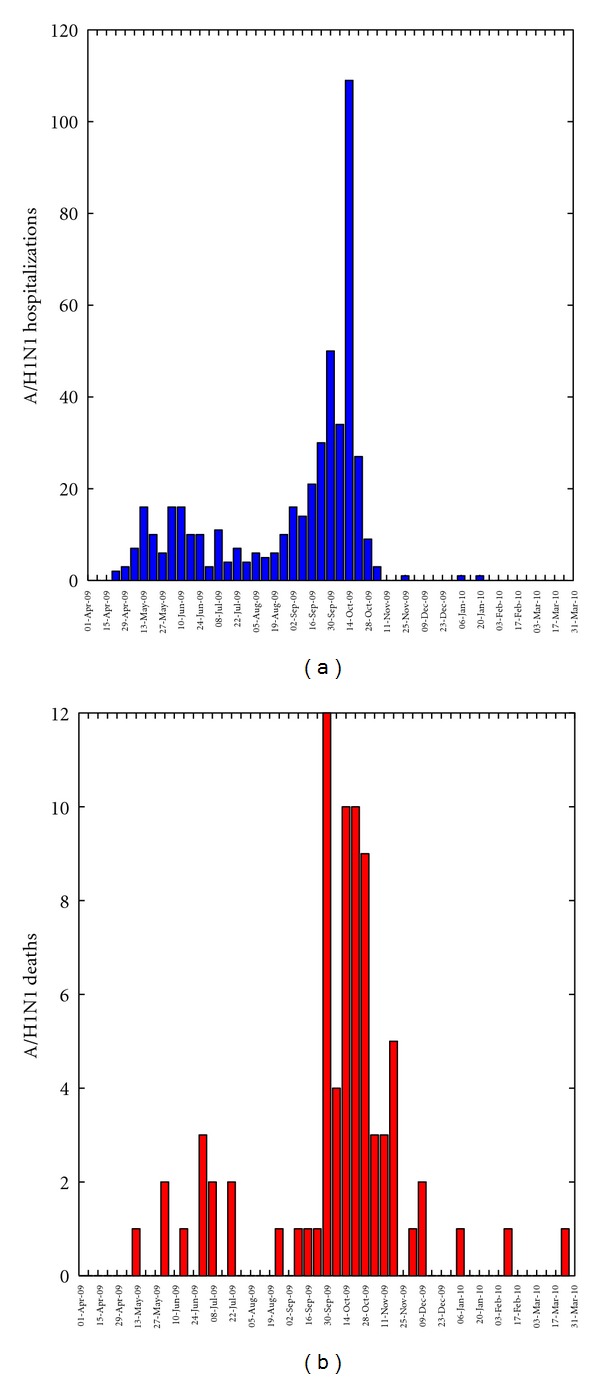
Weekly number of new A/H1N1 influenza inpatients and deaths by date of notification to Maricopa County from April 1, 2009 to March 31, 2010.

The characteristics of A/H1N1 influenza hospitalized patients and deaths with complete case investigations by pandemic wave are shown in Table 1, and the temporal profile of hospitalizations and deaths is illustrated in Figure 1. Of the 609 patients investigated by the MCDPH between April 2009 and March 2010, there were 532 hospitalized patients who recovered, 65 inpatient deaths, and 12 at-home deaths. The number of laboratory-confirmed A/H1N1 hospitalizations (deaths) reported in the spring and fall waves were 144 (11) and 388 (66), respectively. The median age among A/H1N1 hospitalizations was 21 years (range: 0–96 y), and the median age among A/H1N1 inpatient deaths was 46 y (range: 0–80 y). The fraction of A/H1N1 hospitalizations among young persons (18–49 y) increased from 26.9% to 35.3% and decreased from 28.4% to 18.9% among seniors (≥50 y) from the spring to the fall wave.

Hospitalization rates were significantly higher among Native Americans (risk ratio (RR)* * = * *6.2; 95%CI: 6.15, 6.21), non-Hispanic Black (RR = 3.84; 95% CI: 3.8, 3.9), and Hispanics (RR = 2.0; 95% CI: 2.0, 2.01) compared to non-Hispanic Whites. The median age among A/H1N1 hospitalized Native Americans was 21 y (range = 0–73 y), among hospitalized non-Hispanic Blacks was 15 y (range = 0–82 y), among Hispanics was 14 y (range = 0–81 y), and among hospitalized non-Hispanic Whites was 35 y (range = 0–96 y). Slightly higher proportions of females than males (55% males, 45% females) were affected both in the spring and fall pandemic waves (Chi-square test, *P* = 0.99, Table 1). Similarly, the proportions of hospitalizations by ethnicity/race groups were not significantly different in the spring versus the fall pandemic waves (Chi-square test, *P* = 0.54, Table 1).

### 3.1. Temporal Patterns of Admission Delays and Neuraminidase Inhibitor Administration

The average admission delay was slightly but significantly higher during the spring wave compared to the fall wave (2.9 d versus 2.4 d, Wilcoxon test, *P* < 0.001). A total of 361 (72.2%) hospitalized patients were treated with antivirals (Table 2). There was a significant change in the pattern of antiviral administration between the spring and fall pandemic waves of 2009. In particular, antiviral administration rates were at 59.2% among hospitalized patients throughout the spring (April 1 to August 15, 2009) and increased significantly later in the pandemic to 74.4% during the fall pandemic wave (Table 2, Chi-square test, *P* < 0.0001). Moreover, antiviral treatment rates were significantly lower during the spring pandemic wave compared to the fall wave for Hispanics (48.3% versus 76.2%, Chi-square test, *P* < 0.0001) and non-Hispanic whites (61% versus 77.8%, Chi-square test, *P* = 0.02). Antiviral treatment for children and young adults increased during the fall pandemic wave as shown in Table 2. No differences by gender, age, and ethnicity/race in the cumulative antiviral administration rates were observed (*P* > 0.29). 

### 3.2. Hospitalized Case Fatality Ratios

We found significant temporal differences in the hCFR based on hospitalized patients (Table 3). The overall hCFR based on hospitalized patients was estimated at 12.2%. The hCFR was higher during the fall pandemic wave compared to the spring wave despite the increased antiviral administration rates during the fall (7.6% versus 13.9%, Chi-square test, *P* = 0.05). Moreover, hCFR among hospitalized patients increased as age group gets older (Chi-square test, *P* < 0.0001) but was not significantly different by gender (Chi-square test, *P* = 0.94). Furthermore, the hCFR estimates increased among non-Hispanic Whites from the spring to the fall pandemic wave (7.7% versus 22.1%, Chi-square test, *P* = 0.02) and increased twofold among persons ≥50 y from the spring to the fall pandemic wave (14.6% versus 32.9%, Chi-square test, *P* = 0.03). The hCFR was significantly higher among inpatients with admission delays >2 days compared to inpatients with shorter admission delays (21.2% versus 5.9%, Chi-square test, *P* < 0.0001).

### 3.3. Multivariate Logistic Regression Analysis

Among inpatients with confirmed A/H1N1, one or more comorbidities were present in 68.3% of A/H1N1 inpatients under 18 years of age and in 77.2% of inpatients 18 years and older. The most common comorbidities among A/H1N1 inpatient deaths were chronic lung disease (55.4%) and immune suppression (53.8%) (Table 4). 

In a fully adjusted multivariate logistic regression analysis of the risk factors for dying from A/H1N1 influenza hospitalization, we found Asian inpatients to be at an increased risk of death (OR = 24.1 (95% CI: 2.5, 234)) after adjusting for all other factors. We also found chronic lung disease (OR = 3.3 (95% CI: 1.5, 7.5)), cancer within the last 12 months (3.8 (95% CI: 1.01, 14.3)), immunosuppression (OR = 4.6 (95% CI: 2.0, 10.6)), fall pandemic wave (OR = 4.5 (95% CI: 1.82, 11.2)), and admission delay (OR = 11.5 (95% CI: 2.4, 55.2)) to be the strongest predictors of death among A/H1N1 inpatients with full adjustment for all other covariates. Of note, a total of 101 inpatient records (19%) had to be excluded for fully adjusted multivariate logistic regression analyses due to missing data.

A simplified model obtained via backward elimination procedure included age groups, ethnicity/race groups where Asian inpatients had a higher risk of death, pandemic wave, chronic lung disease, cancer within the last 12 months, immunosuppression, and admission delay as shown in Table 5. Antiviral treatment was dropped from the final model as it lacked statistical significance. The model fit to the data was not rejected according to the Hosmer-Lemeshow test statistic (*P* = 0.48). 

## 4. Discussion

Although a number of studies have shed light on the risk factors associated with severe outcomes of 2009 A/H1N1 influenza infections in different populations (e.g., [4–8]), analyses of severity outcomes according to ethnicity/race groups are scarce [9, 10]. Here, we have analyzed 532 A/H1N1 hospitalizations and 77 A/H1N1 deaths in Maricopa County as collected by a prospective surveillance system put in place by the Maricopa County Department of Public Health during April 2009 to March 2010. Our data comprises two main pandemic waves in the spring and fall in Maricopa County, which were associated with high hospitalization rates particularly among racial and ethnic minority groups (Native Americans, non-Hispanic Blacks, and Hispanics). Moreover, we found Asian inpatients to be at a higher risk of death compared to other ethnic/race groups after adjustment for age, gender, antiviral treatment, admission delays, and comorbidities. Our findings also suggest a higher risk of death among A/H1N1 hospitalizations during the major fall pandemic wave after adjusting for factors other than intrinsic changes in virus characteristics. In particular, our results underscore the impact of admission delays and underlying medical conditions particularly immune suppression, chronic lung disease, and cancer within the last 12 months in increasing the risk of death following A/H1N1 hospitalization after adjusting for other covariates.

We found risk of death among A/H1N1 inpatients to be significantly higher during the fall pandemic wave compared to the spring pandemic wave after adjustment for demographic, antiviral treatment, and comorbidities. We note that this result is in contrast with an analysis of the risk of death among hospitalized cases during the summer and fall 2009 A/H1N1 pandemic waves in England [11]. Other factors could have contributed to this result, for example, hospitalization of A/H1N1 cases becoming more selective as the pandemic progressed—that is, only the most severe cases were hospitalized during later months which is in line with the increase in antiviral treatment during the fall wave (Table 2). Overall rates of antiviral administration among hospitalized patients were high during the entire pandemic period in Maricopa County during the 2009 pandemic and are in close agreement with US national estimates (50–82%) [7, 12, 13].

Hospitalization rates among Native Americans, non-Hispanic Blacks, and Hispanics were 2–6 times higher than those observed among non-Hispanic Whites, which is in agreement with an analysis of hospitalized patients during the 2009 A/H1N1 influenza pandemic in Wisconsin, New Mexico, and the US overall [9, 14, 15]. While Asians experienced the lowest A/H1N1 hospitalization rates among ethnic/race groups, this group showed a statistically significant increased risk of death in the hospital (OR = 23.3) after adjustment for other covariates. We note that the corresponding odds ratio for the risk of death among A/H1N1 Asian inpatients lacks precision (wide confidence interval) due to the small number of Asian cases in our sample. Lack of surveillance data, particularly among Asian Americans and Native Hawaiians/Pacific Islanders, has been previously noted, and a smaller influenza burden among Asian Americans and Native Hawaiians/Pacific Islanders has been suggested [15]. Our unexpected findings could be explained as a result of differences in health-care seeking behavior among ethnic/race groups where only the most severe A/H1N1-positive Asian infected cases sought health care services during the pandemic period. 

We note that in our sample 68.3% of hospitalized patients <18 years of age with A/H1N1 infection had one or more comorbidities, which is substantially higher to that reported among A/H1N1 inpatients in Wisconsin (25%) during the 2009 A/H1N1 influenza pandemic [9]. In line with previous studies [16–19], immunosuppression (OR = 4.5 (95% CI: 2.1, 9.7)) was a significant risk factor of mortality among A/H1N1 inpatients in an adjusted multivariate logistic regression analysis. Similarly, chronic lung disease (OR = 3.5) and cancer within the last 12 months (OR = 4.3) were associated with an increase risk of death among A/H1N1 inpatients, which is also in agreement with previous studies [15, 17, 20].

We did not find diabetes to be significantly associated with risk of death among A/H1N1 inpatients. In contrast, previous studies have identified diabetes to be significantly associated with death from pandemic A/H1N1 influenza [16, 21]. Obesity information in our data was missing in over 30% of inpatient records, and, hence, it was excluded from the multivariate logistic regression analysis. Several studies have supported a link between obesity and increased risk of death with seasonal influenza [22] and 2009 pandemic A/H1N1 influenza [8, 21, 23–27]. 

Treatment with antivirals was not found to be significantly associated with a reduced risk of death after adjustment for other covariates. Nevertheless, data on antiviral treatment was obtained retrospectively by examining medical records, but the date of the start of treatment relative to the onset of symptoms was not available for analysis. By contrast, delay in admission >2 days was significantly associated with an increased risk of death (OR = 4.6) in line with previous studies [5, 28–36]. 

Our study has several strengths and limitations worth noting. We used detailed hospitalization data from an enhanced surveillance system that covered all hospitals in the county. However, we included in our analysis only those hospitalizations for which an investigation was conducted (about 50% of all A/H1N1 influenza hospitalizations). Our individual-level clinical data allowed us to assess the effect of ethnic/race groups, age, gender, and underlying comorbidities on the risk of death among A/H1N1 influenza inpatients in a multivariate logistic regression framework. Moreover, risk factor data were recorded in most medical records with 2–4% of inpatient records missing comorbidities except for cardiac disease, which was missing 9.3% of records, and obesity was not included in our analysis as it was missing in >30% inpatient records. 

In summary, we found that Native Americans, non-Hispanic Blacks, and Hispanics experienced significantly higher A/H1N1 hospitalization rates than non-Hispanic Whites, and the risk of death among A/H1N1 inpatients was statistically higher among Asian inpatients compared to other racial and ethnic groups after appropriate adjustment for demographic and medical factors. More studies are needed to better understand potential differential mortality impact of A/H1N1-related hospitalizations among different ethnicity/race groups. Our findings also underscore the role of admission delays, immunosuppression, cancer within the last 12 months, and chronic lung disease on mortality associated with 2009 A/H1N1 pandemic influenza. Providers should recommend influenza vaccination to patients with these medical conditions. In addition, when these high-risk patients do become hospitalized, providers should be aware of their increased risk of fatal outcomes. 

## Figures and Tables

**Table 1:** Characteristics of A/H1N1 influenza hospitalizations and deaths by pandemic wave, Maricopa County, April 1, 2009 to March 31, 2010.

Variable	Total	Pandemic wave		*P* value^a^ (degrees of freedom)
		Spring	Fall	
Age (years)				
<18	246/546 (45.1)	62/144 (43.1)	184/402 (45.8)	0.045 (2)
18–49	183/546 (33.5)	41/144 (26.9)	142/402 (35.3)	
≥50	117/546 (21.4)	41/144 (28.4)	76/402 (18.9)	
Race/ethnicity				
Hispanics	206/546 (37.7)	60/144 (41.7)	146/402 (36.3)	0.54 (5)
Whites, non-Hispanic	207/546 (37.9)	52/144 (36.1)	155/402 (38.6)	
Black, non-Hispanic	62/546 (11.4)	13/144 (9.0)	49/402 (12.2)	
Native American	34/546 (6.2)	9/144 (6.2)	25402 (6.2)	
Asian	5/546 (0.9)	0/144 (0.0)	5/402 (1.2)	
Other/unknown	32/546 (5.9)	10/144 (6.9)	22/402 (5.5)	
Gender				
Female	303/546 (55.5)	80/144 (55.6)	223/402 (55.5)	0.99 (1)
Male	243/546 (44.5)	64/144 (44.4)	179/402 (44.5)	
Patients according to severity				
ICU	153/519 (29.5)	41/140 (29.3)	112/379 (29.6)	0.95 (1)
Mechanical ventilation	106/522 (20.3)	28/144 (19.4)	78/378 (20.6)	0.76 (1)
Deaths	77/546 (14.1)	11/144 (7.6)	66/402 (16.4)	0.009 (1)

Wave 1 (spring) refers to April 1 through August 15, 2009; fall wave refers to August 16, 2009 through March 31, 2010. Data are percentages of cases unless otherwise specified.

^a^Determined by the Chi-square test statistic. *P* values for age and race/ethnicity obtained from group wise comparisons, while *P* values for gender and severity comparisons correspond to each category.

**Table 2:** Rates of antiviral administration (mean and 95% confidence intervals) among A/H1N1 influenza inpatients by pandemic wave, Maricopa County, April 1, 2009 to March 31, 2010.

Variable	Total	Pandemic wave		*P* value^a^
		Spring	Fall	
Number of patients that received antivirals (% of total A/H1N1 cases)	361/500 (72.2)	84/142 (59.2)	277/358 (74.4)	<0.0001
Race/ethnicity				
Hispanics	128/190 (67.4)	29/60 (48.3)	99/130 (76.2)	<0.0001
Whites, non-Hispanic	136/186 (73.1)	31/51 (60.8)	105/135 (77.8)	0.02
Black, non-Hispanic	46/59 (78.0)	12/13 (92.3)	34/46 (73.9)	0.16
Native American	24/30 (80.0)	6/8 (75.0)	18/22 (81.8)	0.68
Asian	4/4 (100)	0/0 (0.0)	4/4 (100.0)	N/A
Gender				
Female	206/280 (73.6)	49/79 (62.0)	157/201 (78.1)	0.006
Male	155/220 (70.5)	35/63 (55.6)	120/157 (76.4)	0.002
Age (years)				
<18	161/231 (69.7)	31/61 (50.8)	130/170 (76.5)	<0.0001
18–49	122/162 (75.3)	22/40 (55.0)	100/122 (82.0)	0.001
≥50	78/107 (72.9)	31/41 (75.6)	47/66 (71.2)	0.62
Patients according to severity				
ICU	111/150 (74.0)	23/41 (56.1)	88/109 (80.7)	0.002
Mechanical ventilation	73/105 (69.5)	13/28 (46.4)	60/77 (77.9)	0.002
Deaths	43/63 (68.3)	5/11 (45.5)	38/52 (73.1)	0.074

Wave 1 (spring) refers to April 1 through August 15, 2009; fall wave refers to August 16, 2009 through March 31, 2010. Data are percentages of cases unless otherwise specified.

^a^Determined by the Chi-square test statistic. *P* values show the univariate comparisons of proportions between spring and fall waves.

**Table 3:** Unadjusted case fatality ratios among A/H1N1 influenza inpatients by pandemic wave, Maricopa County, April 1, 2009 through March 31, 2010.

Variable	Overall hospitalized case fatality ratio	Pandemic wave		*P* value^a^
		Spring	Fall	
Total A/H1N1 deaths (% of total A/H1N1 cases)	65/532 (12.2)	11/144 (7.6)	54/388 (13.9)	0.049
Race/ethnicity				
Hispanics	18/202 (8.9)	5/60 (8.3)	13/142 (9.2)	0.85
Whites, non-Hispanic	36/197 (18.3)	4/52 (7.7)	32/145 (22.1)	0.021
Black, non-Hispanic	5/62 (8.1)	2/13 (15.4)	3/49 (6.1)	0.31
Native American	4/34 (11.8)	0/9 (0.0)	4/25 (16.0)	0.20
Asian	2/5 (40.0)	0/0 (0.0)	2/5 (40.0)	N/A
Gender				
Female	36/297 (12.1)	5/80 (6.2)	31/217 (14.3)	0.060
Male	29/235 (12.3)	6/64 (9.4)	23/171 (13.5)	0.40
Age (years)				
<18	11/243 (4.5)	2/62 (3.2)	9/181 (5.0)	0.57
18–49	24/175 (13.7)	3/41 (7.3)	21/134 (15.7)	0.17
≥50	30/114 (26.3)	6/41 (14.6)	24/73 (32.9)	0.034

Wave 1 (spring) refers to April 1, 2009 through August 15, 2009; fall wave refers to August 16, 2009 through March 31, 2010. Data are percentages of cases unless otherwise specified.

^a^Determined by the Chi-square test statistic. *P* values show the univariate comparisons of proportions between spring and fall waves.

**Table 4:** Frequency of risk factors for A/H1N1 inpatients, Maricopa County, April 1, 2009 to March 31, 2010.

Risk factors	A/H1N1 inpatients	A/H1N1 fatal	A/H1N1 hospitalization versus
			A/H1N1 death odds ratio (95% CI)^a^
Asthma	127/449 (28.3)	16/64 (25.0)	0.73 (0.32, 1.7)
Chronic lung disease	101/447 (22.6)	36/65 (55.4)	3.3 (1.5, 7.5)
Cardiac disease	80/419 (19.1)	23/65 (35.4)	1.33 (0.54, 3.3)
Metabolic disease	71/450 (15.8)	21/65 (32.3)	3.35 (0.55, 20.5)
Diabetes	61/451 (13.5)	19/65 (29.2)	0.3 (0.04, 2.11)
Kidney disease	34/453 (7.5)	11/64 (17.2)	0.96 (0.31, 3.01)
Cancer last 12 months	16/455 (3.5)	13/65 (20.0)	3.8 (1.01, 14.3)
Immune suppression	93/458 (20.3)	35/65 (53.8)	4.6 (2.0, 10.6)
Neurological disease	80/455 (17.6)	20/65 (30.8)	1.7 (0.72, 4.1)

^a^Risk of death among A/H1N1 inpatients adjusted by age, gender, ethnicity/race, pandemic wave, and antiviral treatment.

**Table 5:** Final logistic regression model of risk of death based on A/H1N1 hospitalizations and obtained via backward elimination procedure.

Risk factors	*P* value	OR (95% CI)^a^
Age groups		
<18 y	0.004	0.27 (0.11, 0.66)
18–49 y	0.83	1.1 (0.47, 2.6)
≥50 y	Ref value	1
Ethnicity/race		
Unknown	0.99	0
Hispanics	0.24	1.65 (0.72, 3.8)
Native American	0.75	1.24 (0.33, 4.7)
Non-Hispanic black	0.54	0.67 (0.20, 2.4)
Asian	0.008	23.3 (2.3, 238.3)
Non-Hispanic white	Ref value	1
Pandemic wave		
Fall wave	0.001	3.94 (1.72, 9.03)
Chronic lung disease	0.001	3.5 (1.66, 7.4)
Cancer within last 12 months	0.02	4.3 (1.3, 14.8)
Immune suppression	0.001	4.0 (1.84, 8.9)
Admission delay	<0.001	4.6 (2.2, 9.5)

^a^Risk of death among A/H1N1 inpatients. The corresponding Hosmer-Lemeshow test statistic for model fit (*P* = 0.48) and the Cox and Snell *R* ^2^ = 0.22.
